# Chemorefractory extranodal nasal-type natural-killer/T-cell lymphoma with great response to pembrolizumab in a young patient: a case report

**DOI:** 10.1186/s13256-021-03079-8

**Published:** 2021-10-26

**Authors:** Fatemeh Adabifirouzjaei, Bharam Khazai, Ghazaleh Azami, Ghazaleh Shoja-e-Razavi

**Affiliations:** 1 Sulpizio Cardiovascular Center, University of California San Diego, San Diego, California USA; 2 Harbor-UCLA Medical Center, University of California Los Angeles, Torrance, CA USA; 3 Internal Medicine Department, School of Medicine, Mashhad University of Medical Sciences, Mashhad, Iran; 4 Hematology and oncology Section, Division of Hematology and Oncology, Internal Medicine Department, School of Medicine, The University of Calgary, Alberta, Canada

**Keywords:** Extranodal NK/T-cell lymphoma, Epstein–Barr virus, l-Asparaginase, High-dose methotrexate, Brentuximab vedotin, Pembrolizumab, Case report

## Abstract

**Introduction:**

Extranodal, natural-killer/T-cell lymphoma of nasal type is a rare but aggressive disease usually presenting as progressive necrotic lesions in the nasal cavity that responds poorly to chemotherapy. In this paper, we report a relapsing, chemorefractory case of extranodal natural-killer/T-cell lymphoma responding to checkpoint inhibitor immunotherapy with pembrolizumab.

**Case presentation:**

A 32-year-old Hispanic woman with a history of recurrent sinusitis and preseptal abscess presented with a hoarse voice, swelling around the right eye, and diplopia. Laryngoscopy showed infiltrating tissue extending to bilateral laryngeal ventricles and false vocal cords. On immunohistochemical examination of laryngeal biopsy, the neoplastic cells showed positivity for CD3 (cytoplasmic), CD7, CD56, granzyme B, CD30, and Epstein–Barr virus-encoded ribonucleic acid (RNA). Extranodal natural-killer/T-cell lymphoma, nasal type, was confirmed. In the absence of distant organ involvement, the decision was to perform chemotherapy with etoposide, ifosfamide, mesna, cisplatin, and dexamethasone (VIPD protocol) followed by concurrent chemoradiation with weekly doses of cisplatin and two cycles of VIPD as adjuvant treatment. However, 1 month after completion of the treatment; disease recurrence was demonstrated. The patient was scheduled to receive salvage chemotherapy with steroid, methotrexate, ifosfamide, L- asparaginase, and etoposide (SMILE) protocol and CD30-targeting monoclonal antibodies. However, the mass was chemorefractory without response to either l-asparaginase-based salvage chemotherapy in combination with high-dose methotrexate or brentuximab vedotin. However, this case of chemorefractory extranodal natural-killer/T-cell lymphoma, nasal type, responded well to the novel drug pembrolizumab, which was able to control the disease.

**Conclusion:**

Checkpoint inhibitors are potential treatment option in selected chemorefractory extranodal natural-killer/T-cell lymphoma, nasal type, cases.

## Introduction

Extranodal, nasal-type natural-killer (NK)/T-cell lymphoma is a rare and aggressive type of lymphoma. Although there is no standard treatment approach for the disease, the preferred approach in the localized disease is chemoradiation and, in advanced cases, combination chemotherapy [1].

## Case presentation

A 32-year-old Hispanic woman, G1P1L1, stay-at-home mom, with a history of recurrent sinusitis and preseptal abscess for 2 years presented with a hoarse voice, swelling around the right eye, and diplopia in July 2018. She was previous a cocaine (occasionally since 2015) user for 2 years, who had quit it 2 years before presenting to the hospital. She had no family history of cancer or hematological diseases. She had a blood pressure of 124/81 mmHg, heart rate of 113 beats per minute, and temperature of 37.1° C on first admission. On physical examination, swelling of the right orbit, including the eyelid, was noted. Nasal examination showed some erythema to the nasal mucosa but no perforated septum and no saddle nose deformity. There was no inflammation in the ears, and the inner ears looked normal with normal tympanic membranes. On neurological examination, limited eye movements on the right side with no ability for abduction or adduction indicating extraocular muscle involvement were detected. Sinus mucosa biopsy showed chronic inflammation featuring heavy perivascular infiltration of mixed lymphoplasmacytic cells and histiocytes. Despite being on prednisone owing to the primary impression of autoimmune nature of the disease, over a period of 3–4 months, there was progression of symptoms causing hoarse voice and further swelling around the right eye, diplopia, and limitation of right eye movement. Complete blood count results revealed 9 × 10^3^ white blood cells with 6 × 10^3^ neutrophils, 2 × 10^3^ lymphocytes, and 1 × 10^3^ monocytes, with no eosinophils and basophils. Hemoglobin level was 14.4 g/L, and platelet count was 328 × 10^3^/µL. The other blood test results were as follows: creatinine 0.75 μmol/L, urea 1.9 mg/dL, aspartate aminotransferase 15 units/L, alanine aminotransferase 26 units/L, alkaline phosphatase 89 units/L, and gamma glutamyl transferase 42 units/L.

Direct laryngoscopy in December 2018 showed infiltrating tissue involving laryngeal ventricles bilaterally extending towards the anterior aspect of false vocal cords. Magnetic resonance imaging (MRI) of the orbit also showed infiltration and enlargement of the right medial rectus muscle.

**Fig. 1.**
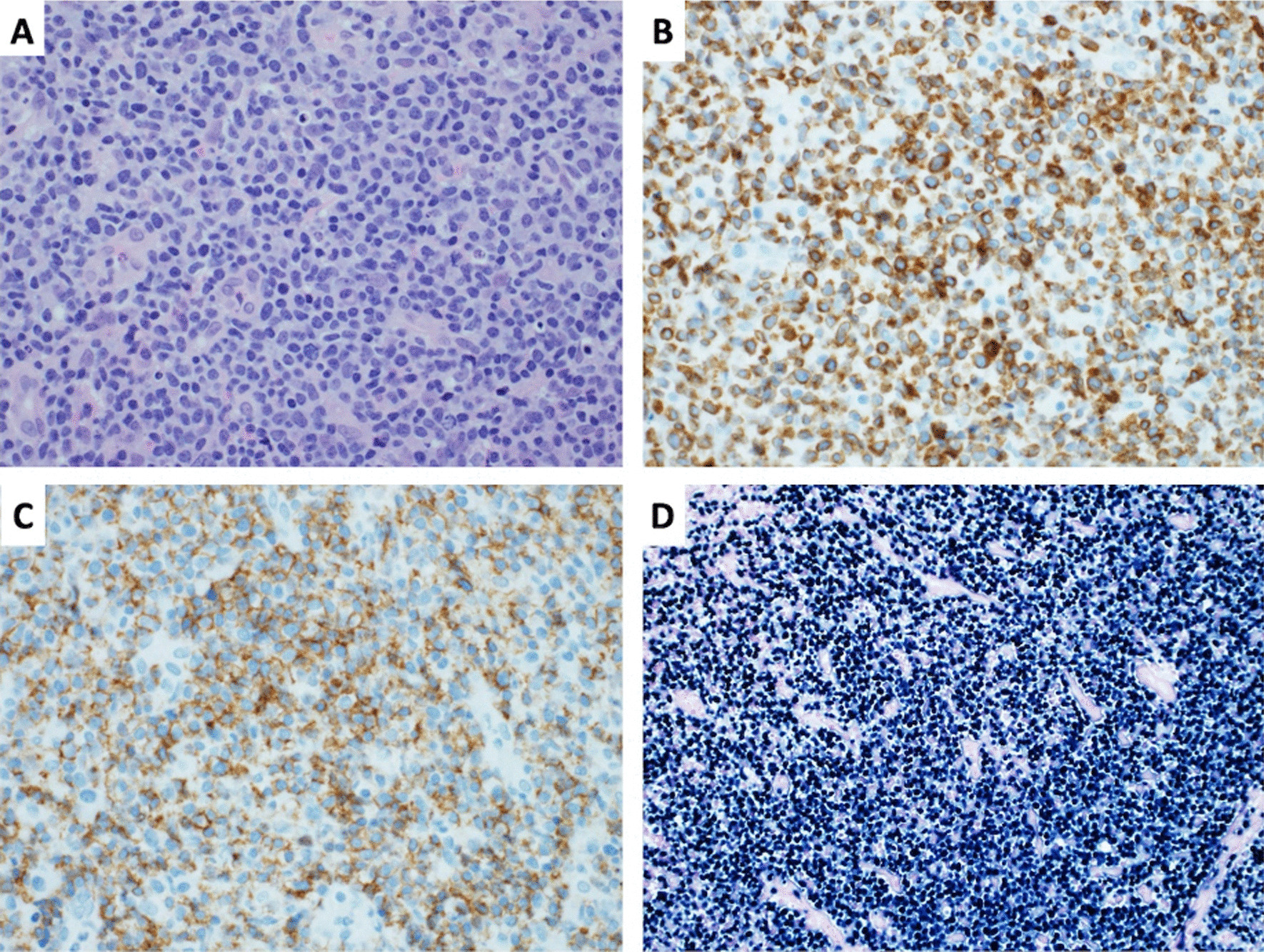
Photomicrographs illustrating the salient features on histomorphology. This figure shows the salient features on histomorphology of the patient’s laryngeal biopsy with **A** hematoxylin and eosin, 40×; **B** CD3 antibody stain demonstrating cytoplasmic (cCD3-epsilon) staining pattern; **C** CD56 antibody stain > 75% of the tumor cells; **D** EBV-encoded small RNA (EBER) by *in situ* hybridization technique

Both laryngeal infiltrating lesions and medial rectus muscle of the right eye were biopsied. On immunohistochemical examination of laryngeal infiltration biopsy, the neoplastic cells showed diffuse positivity for cluster of differentiation (CD)3 (cytoplasmic), CD7, CD56, granzyme B, CD30, and multiple myeloma oncogene-1 (MUM1). Results were negative for CD5, CD4, CD8, CD10, CD20, and PAX5. *In situ* hybridization study was positive for Epstein–Barr virus (EBV)-encoded ribonucleic acid (EBER). Monoclonal T-cell receptor (TCR) gene arrangement was detected by polymerase chain reaction (PCR) (Fig. 1).

**Fig. 2.**
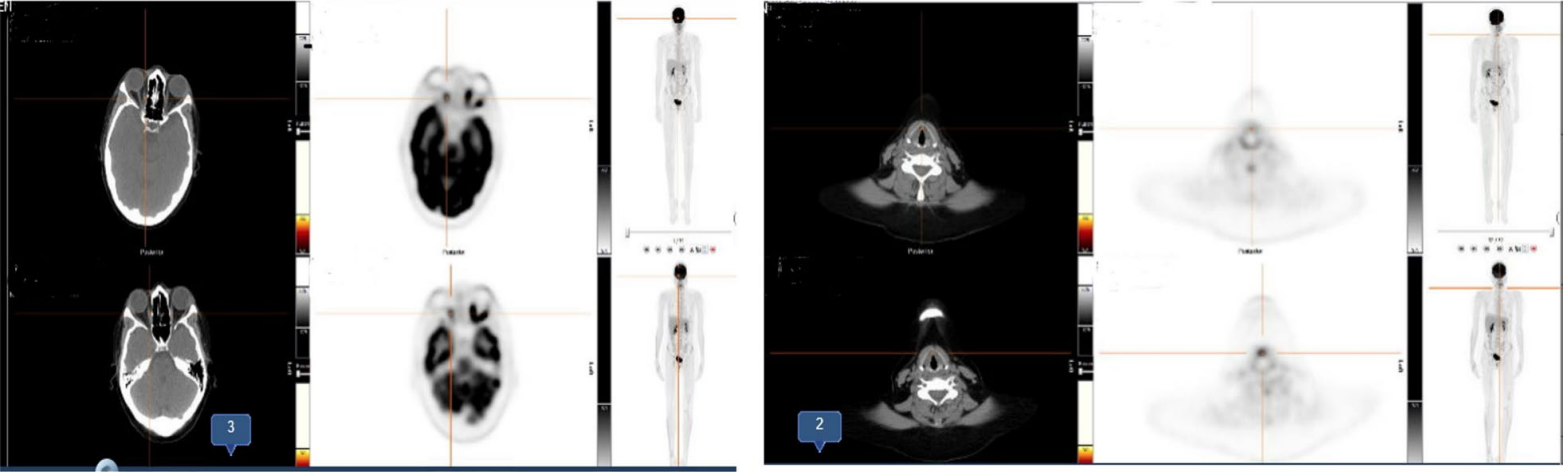
Fluorodeoxyglucose- positron emission tomography (FDG-PET) images of patient, before starting pembrolizumab. FDG PET shows a discrete focus of hypermetabolism in the medial rectus muscle on the right corresponding to the area of greatest thickening, suggestive of residual lymphoma as physiologic activity in the other extraocular muscles on the right is absent. There is a focal area of hypermetabolism within the anterior soft tissues adjacent to the intrinsic laryngeal musculature in keeping with viable lymphoma in laryngeal area

Nasal-type extranodal NK/T-cell lymphoma was confirmed. Right medial rectus muscle biopsies, however, were negative for lymphoma with a confirmed negative TCR by PCR. Investigation for human immunodeficiency virus (HIV) and human T-cell leukemia/lymphoma virus (HTLV1) was negative. Bone marrow aspiration and biopsy were also negative. Fluorodeoxyglucose (FDG)-positron emission tomography (PET) computed tomography (CT) scan at the time of diagnosis (Fig. 2) showed a discrete hypermetabolic focus in the right medial rectus muscle corresponding to the area of greatest thickening. Mild residual mucosal thickening was seen in the maxillary antra and nasal mucosa. A focal hypermetabolic area was noted in the anterior laryngeal soft tissues adjacent to the intrinsic laryngeal muscles indicating viable lymphoma. There was no metabolic activity in the rest of body based on the PET CT results.

Since the lymphoma was localized to the laryngeal area with no systemic involvement, the patient received one cycle of chemotherapy in February 2019 with VIPD protocol containing etoposide (100 mg/m^2^, intravenous infusion, days 1–3), ifosfamide (1200 mg/m^2^, intravenous infusion, once a day, days 2–4), mesna (330 mg/m^2^ of mesna was given before ifosfamide, and 480 mg/m^2^ was given 4 and 8 hours after infusion for 3 subsequent days , intravenous infusion, three times a day), cisplatin (33 mg/m^2^, intravenous infusion, weekly), and dexamethasone (40 mg, once daily, days 1–4, with G-CSF support). Two more cycles of VIPD were also scheduled in April and May of 2019, after the completion of chemoradiation. FDG-PET CT scan after completion of treatment was done in June 2019 and did not show convincing metabolic activities to suggest residual viable lymphoma. The previously noted hypermetabolic soft-tissue nodule at the anterior aspect of the larynx was resolved.

However, within a month after the PET-CT scan featuring complete metabolic response, the patient presented with an enlarged right-sided submandibular infiltrative mass. CT scan showed asymmetric mass in submandibular glands area that was bulkier in the right and demonstrated heterogeneous enhancement in favor of recurrent disease. Orbital CT scan also reported an asymmetric soft-tissue mass and stranding involving the right preseptal and periorbital fat predominantly at the inferior aspect of the periorbital tissue.

A repeat biopsy from the right lower eyelid confirmed involvement by the previously diagnosed nasal-type EBER-positive extranodal NK/T-cell lymphoma.

Systemic investigation did not show any distant involvement. The patient was scheduled to receive salvage chemotherapy with SMILE protocol in August and September 2019 [oral methotrexate 2 g/mg, orally, on day 1, leucovorin 15 mg, intravenous infusion, four times a day, on days 2–4, ifosfamide 1500 mg/m^2^, intravenous infusion, on days 2–4, mesna 300 mg/m^2^, intravenous infusion, once daily, on days 2–4, dexamethasone 40 mg, daily, on days 2–4, etoposide 100 mg/m^2^, intravenous infusion, on days 2–4, l-asparaginase 6000 U/m^2^, intramuscular, on days 8, 10, 12, 14, 16, 18, and 20, granulocyte colony-stimulating factor (GCSF) support from day 6 and discontinued if the leukocyte count exceeded 5000/μL]. Tumor cells also showed CD30 expression; thus, CD30-targeting monoclonal antibody, brentuximab vedotin (1.8 mg/kg every 3 weeks) was added to the treatment in October 2019. Salvage chemotherapy with SMILE protocol was complicated with neutropenia and thrombocytopenia. In addition, brentuximab vedotin induced grade 3 sensory and motor neuropathies in both lower extremities while the disease did not respond to the treatment.

**Fig. 3.**
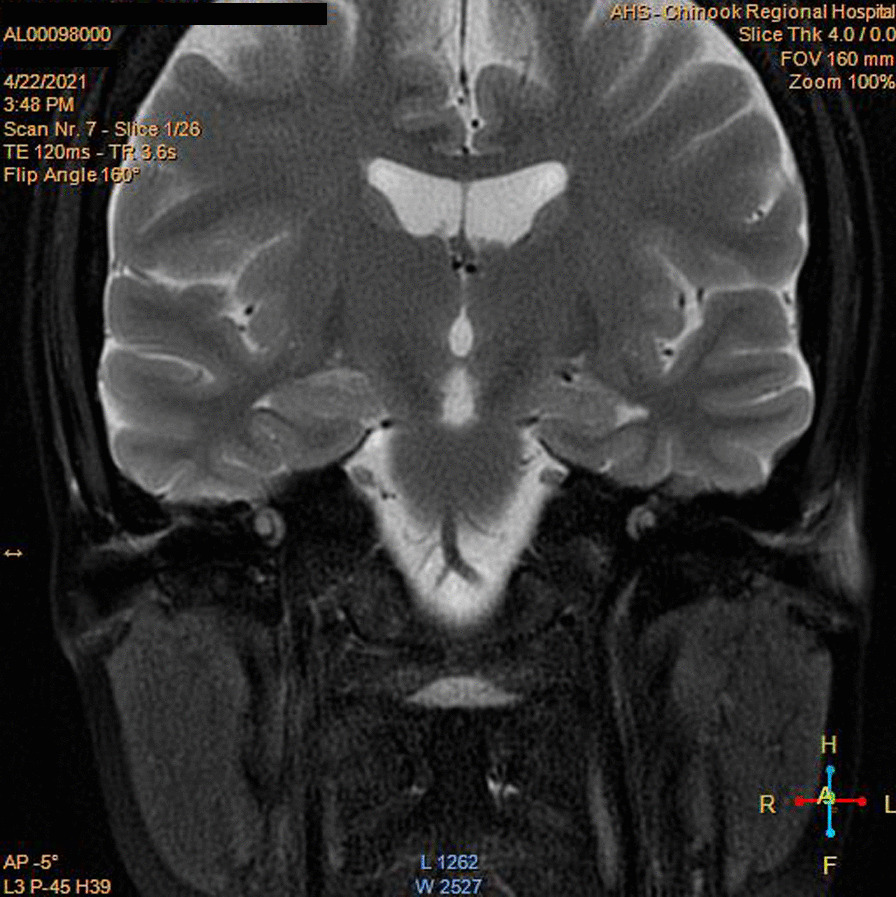
Orbit and cervical MRI of patient during pembrolizumab therapy. Magnetic resonance imaging (MRI) show symmetric appearance of the larynx, vocal cords, and hypopharynx soft tissues. Submandibular glands have a fairly symmetric appearance. Previously seen abnormality on the right is not present today. There is no evidence of identified adenopathy or abnormal soft-tissue enhancement

Subsequently, pembrolizumab was given at the dose of 100 mg every 3 weeks starting December 2019 with a clinical response after the third cycle of treatment, and the patient remained disease-free on this treatment. Orbital and cervical MRI figures during this therapy are shown in Fig. 3. The patient is currently alive and disease-free with grade 2 motor neuron toxicity on the left ankle that has remained unchanged since chemotherapy with brentuximab vedotin. The swelling of the eye and voice changes are completely back to normal. The patient’s Eastern Cooperative Oncology Group (ECOG) performance status is 1. Fatigue and mild nausea are ongoing adverse events that she has been experiencing. Both symptoms are considered to be grade 2 and are not impairing the patient’s quality of life. Her last follow-up in the cancer center was on 10 June 2021.

## Discussion

NK/T-cell lymphomas almost exclusively involve non-nodal sites and are, hence, called extranodal NK/T-cell lymphoma (ENKTL). In nearly 80% of patients, the initial presentation sites include the oropharynx, the Waldeyer’s ring, and the upper aerodigestive tract [1]. The pathogenesis of ENKTL remains unknown; however, recent genomic data have revealed a complex interaction between the Janus kinase (JAK)/signal transducer and activator of transcription (STAT) pathways, epigenetic dysregulation, disrupted nuclear factor kappa-light-chain-enhancer of activated B cells (NFkB), and mitogen-activated protein kinase (MAPK) pathways as a possible pathophysiology behind this lymphoma [2].

Histological confirmation of the disease is essential and should reveal neoplastic lymphoid cells associated with plasma cells, polymorphonuclear neutrophils, and histiocytes presenting in an angiocentric pattern [3]. Due to expression of cytokines and cytotoxic molecules by the tumor cells, fibrinoid or coagulative necrosis may be seen in these patients. CD56 or EBER staining is helpful in this setting. The lymphoma cells express CD2 and CD3e, but usually not CD3s or other T-cell markers (CD4, CD5, and CD8) [4].

EBV+ T- and NK-cell lymphoma frequently expresses CD30.These cells always express EBER and cytotoxic molecules (TIA-1, perforin, or granzyme B) [5].

Plasma EBV DNA concentration is proportional to the tumor bulk; therefore, quantification of EBV DNA can be useful for both baseline prognostication and future monitoring [6]. ENKTL is F18-FDG avid, and PET-CT is now considered a standard imaging modality for this type of lymphoma. Conventional lymphoma treatments such as CHOP are ineffective in ENKTL [7]. Either chemotherapy followed by radiotherapy or concurrent chemoradiotherapy can be considered as the first-line treatment for stage I/II ENKTL patients. l-asparaginase-containing regimens are efficacious for the treatment of stage III/IV ENKTL, and the SMILE regimen is the standard therapy [8].

Novel immunotherapies are considered for advanced-stage or relapsed/refractory ENKTCL (Table 1) [9–11]. Given that programmed death ligand 1 (PD-L1) is expressed in a substantial portion of ENKTL, it is an important biomarker that is widely used for patient selection for immunotherapy [12]. Pembrolizumab blocks PD-L1 owing to EBV-driven overexpression of the latent membrane proteins [latent membrane protein 1 (LMP1) and LMP2] with activation of the NF-κB/MAPK pathways [13]. Patients failing l-asparaginase regimens and allogeneic hematopoietic stem cell transplantation (HSCT) have been successfully treated with pembrolizumab [14]

**Table 1:** Cases previously treated with programmed cell death protein 1- programmed death ligand 1 blockers

Study	Sex	Age	Relapse sites	Stage	ECOG	Previous treatment	Treatment	Response	Outcome	Survival months
Kwong *et al*. [1]	M	68	Calf skin	IE	1	SIMPLE	Pembrolizumab	Metabolic and clinical CR	Continuous CR	4+
	M	49	Liver, spleen	IV	1	SMILE	Pembrolizumab	PR	Died of infection	6
	M	38	Nasopharynx, hard palate	IV	2	m-BACOO	Pembrolizumab	Clinical, molecular, and metabolic CR	Continuous CR	10+
	M	50	Liver	IV	3	GELOX	Pembrolizumab	Morphological and radiological CR	Continuous CR	8+
	M	31	Nasal cavity, liver	IV	2	SMILE	Pembrolizumab	Could not be assessed	Died of sepsis consequent on gastric bleeding	2
	M	35	Lung, esophagus	IV	2	SMILE	Pembrolizumab	CR	Continuous CR	9
	M	51	Liver	IV	1	SMILE	Pembrolizumab	CR	Continuous CR	3
Chan *et al*. [2]	M	59	Cerebellum and dorsal midbrain	IV	3	SIMPLE	Nivolumab	Clinical and radiological CR	Died of infection	1
	M	43	Jejunum, mesenteric lymph node	II	0	SMILE	Nivolumab	Radiological CR	Continuous CR	42
	F	80	Skin, lymph node, liver, spleen, marrow	IV	4	SIMPLE	Nivolumab	Pathological CR	Died of infection	3

CR, complete response; ECOG, Eastern Cooperative Oncology Group; GELOX, gemcitabine, l-asparaginase, and oxaliplatin; m, male; m-BACOD, methotrexate, bleomycin, doxorubicin, cyclophosphamide, vincristine, and dexamethasone; SIMPLE, dexamethasone, ifosfamide, gemcitabine, cisplatin, l-asparaginase, and etoposide; SMILE, dexamethasone, methotrexate, ifosfamide, l-asparaginase, and etoposide

## Conclusion

We presented a case of a 32-year-old woman with a history of recurrent sinusitis and preseptal abscess who presented with pansinusitis. Laryngoscopy showed infiltrating tissue extending to bilateral laryngeal ventricles and false vocal cords. On immunohistochemical examination of laryngeal biopsy, ENKTCL-NT was confirmed. The patient was refractory to both VIPD and SMILE protocols, and CD30-targeting monoclonal antibodies. The patient responded well to pembrolizumab despite rapid progression after chemoradiotherapy. Unlike salvage chemotherapy and CD30-targeted immunotherapy with grade 3 toxicities, immunotherapy with pembrolizumab was tolerated well with minimal grade 1–2 toxicity. This case suggests checkpoint inhibitors as a potential treatment option in selected chemorefractory ENKTCL-NT cases.

## Patient perspective

The patient was very happy with being switched from high-dose chemotherapy that needed hospitalization to outpatient immunotherapy. In fact, she has tolerated immunotherapy with minimal side effects, mostly mild fatigue. She has been very happy with the results, including the overall disease control as well as resolution of the eye swelling and the change of voice back to normal.

## Data Availability

Not applicable.

## Abbreviations

EBER: Epstein–Barr virus-encoded RNA
EBV: Epstein–Barr virus
ENKTCL: Extranodal natural-killer/T-cell lymphoma
ENKTCL-NT: Extranodal natural-killer/T-cell lymphoma, nasal type
NHL: Non-Hodgkin lymphoma
